# Quantitative digital pathology enables automated and quantitative assessment of inflammatory activity in patients with autoimmune hepatitis

**DOI:** 10.1016/j.jpi.2024.100372

**Published:** 2024-03-12

**Authors:** Piotr Socha, Elizabeth Shumbayawonda, Abhishek Roy, Caitlin Langford, Paul Aljabar, Malgorzata Wozniak, Sylwia Chełstowska, Elzbieta Jurkiewicz, Rajarshi Banerjee, Ken Fleming, Maciej Pronicki, Kamil Janowski, Wieslawa Grajkowska

**Affiliations:** a Department of Gastroenterology, Hepatology, Nutritional Disorders and Pediatrics, The Children's Memorial Health Institute, Warsaw, Poland; b Perspectum Diagnostics, Oxford, UK; c Department of Pathology, The Children's Memorial Health Institute, Warsaw, Poland; d Department of Diagnostic Imaging, The Children's Memorial Health Institute, Warsaw, Poland

**Keywords:** Digital pathology, Inflammation, Chronic liver disease, Autoimmune hepatitis

## Abstract

**Background:**

Chronic liver disease diagnoses depend on liver biopsy histopathological assessment. However, due to the limitations associated with biopsy, there is growing interest in the use of quantitative digital pathology to support pathologists. We evaluated the performance of computational algorithms in the assessment of hepatic inflammation in an autoimmune hepatitis in which inflammation is a major component.

**Methods:**

Whole-slide digital image analysis was used to quantitatively characterize the area of tissue covered by inflammation [Inflammation Density (ID)] and number of inflammatory foci per unit area [Focal Density (FD)] on tissue obtained from 50 patients with autoimmune hepatitis undergoing routine liver biopsy. Correlations between digital pathology outputs and traditional categorical histology scores, biochemical, and imaging markers were assessed. The ability of ID and FD to stratify between low-moderate (both portal and lobular inflammation ≤1) and moderate-severe disease activity was estimated using the area under the receiver operating characteristic curve (AUC).

**Results:**

ID and FD scores increased significantly and linearly with both portal and lobular inflammation grading. Both ID and FD correlated moderately-to-strongly and significantly with histology (portal and lobular inflammation; 0.36≤R≤0.69) and biochemical markers (ALT, AST, GGT, IgG, and gamma globulins; 0.43≤R≤0.57). ID (AUC: 0.85) and FD (AUC: 0.79) had good performance for stratifying between low-moderate and moderate-severe inflammation.

**Conclusion:**

Quantitative assessment of liver biopsy using quantitative digital pathology metrics correlates well with traditional pathology scores and key biochemical markers. Whole-slide quantification of disease can support stratification and identification of patients with more advanced inflammatory disease activity.

## Introduction

Liver Inflammation is a common characteristic of several chronic liver diseases including viral hepatitis, autoimmune hepatitis (AIH) and non-alcoholic steatohepatitis (NASH). Typically, liver enzymes, aminotransaminases (alanine and aspartate), are used as surrogate markers to guide disease monitoring resulting from inflammation. However, the additional morphological information, including an assessment of fibrosis, provided by liver biopsy means that it remains a necessary additional assessment when characterising disease severity^10^ and forms part of the requirements to confirm diagnosis of several chronic liver diseases. Nevertheless, due to the subjective nature of histopathology assessment, liver biopsy has well-documented limitations which make it an imperfect standard.^7^^,^^12^^,^^28^

To overcome the subjectivity and limitations associated with traditional histopathology reporting, computer-assisted digital image analysis technologies are being developed to quantitatively and objectively assess histological features.^6^^,^^9^^,^^14^^,^^15^^,^^29^ Although quantitative digital pathology is a recent development, the use of computational methods [including Artificial Intelligence (AI)] to support medical image evaluation is well-established in other fields, including the detection of cancer on computerized tomography (CT) images and stroke risk prediction using magnetic resonance imaging (MRI).^20^ Developments in slide scanning technology, have resulted in whole-slide images (WSIs) of sufficient quality for pathologists to effectively review digital biopsy cases.^1^ To encourage this, regulatory bodies such as the U.S. Food and Drug Administration are also recognizing the importance of digital pathology, issuing 510(k) approvals for WSI viewing platforms (PAIGE, Phillips, Leica), and *de novo* marketing authorisation for clinical AI tool for diagnosis of prostate cancer.^21^

Biopsy grading is a key biomarker endpoint for clinical trials for therapies to treat different liver diseases.^18^^,^^24^ As inflammation plays a key role in the progression of most chronic liver diseases, correctly quantifying the inflammatory burden in the organ under investigation is of paramount importance. However, when compared to the assessment of other histological features, the inter-reader variability between pathologists is generally higher.^7^ Not only is this problematic in routine clinical management, but it poses a large hurdle in assessing the efficacy of a new therapy in clinical trials.^7^ Studies have shown quantification of inflammatory activity using digital pathology algorithms to differentiate between grades of inflammation, albeit using immunohistochemical staining for CD45.^15^ Although these results highlighted the strong potential of digital pathology algorithms to aid pathologists in their evaluation of chronic liver disease biopsies, it was not possible to differentiate between lobular and portal inflammation. Hematoxylin and Eosin (H&E) staining is the conventional method used to assess both portal and lobular inflammation in histological tissue in both clinical practice and clinical trials.^5^^,^^8^^,^^22^ Therefore, there is a need to assess the utility of digital pathology-derived algorithms on histology images of tissue stained with H&E to support more disease specific assessments.

In this study, we evaluated the performance of quantitative digital pathology-derived algorithms to quantify inflammation on H&E slides from a well-characterized cohort of pediatric patients with AIH. Our aim was to identify and quantify hepatic inflammatory areas and to evaluate their association with the corresponding consensus pathologists´ grading scores. In addition, we sought to evaluate the use of these scores to differentiate between grades of inflammation.

## Methods

### Study design and subjects

A longitudinal prospective observational study enrolled 50 pediatric patients aged between 6 and 18 (mean: 14 ± 3) with biopsy confirmed AIH due to have a liver biopsy as part of routine clinical care. Patients were recruited into the study entitled *Kids4Life: Assessing Kids for Liver Inflammation and Fibrosis using non-invasive MRI* which was registered as a clinical trial (NCT03198104) and sponsored by the Eureka Eurostars 2 Grant (E!10124). All patients were under the care of hepatologists at the Children’s Memorial Health Institute in Warsaw (IPCZD) and underwent a research non-contrast MRI scan alongside their routine care assessments, including serum liver biochemistry and liver biopsy. Informed consent and assent (where required) was provided by all subjects and their caregivers, respectively prior to their participation in the study. The study protocol conformed to the ethical guidelines of the 1975 Declaration of Helsinki and received ethical approval (11/KBE/2016) in Poland.

### Histopathology assessment

All histopathology reads were performed in consensus by two experienced liver pathologists (as part of standard clinical care at IPCZD). Liver tissue was obtained using a 16G Menghini needle. Percutaneous liver biopsies were performed under ultrasound guidance after which the obtained sample was assessed for lobular and portal inflammation (using the Batts and Ludwig score^2^), fibrosis using the Ishak score,^10^ steatosis (and parenchymal fat percentage), ballooning, cholestasis, diffuse post-necrotic scarring, collagen proportion, and necrosis.^2^^,^^3^

### Digital image analysis

After histological scoring, original biopsy glass slides were digitized into WSIs for subsequent analysis using a 20× magnification objective and a calibrated camera (1 pixel is 0.456 × 0.456 μm). The images were stored in an 8-bit RGB colour pyramidal format. Digital image analysis was performed at the highest resolution level using software developed in-house and trained and validated using data from the multi-organ nuclei segmentation challenge (MoNuSeg)^13^ and triple negative breast cancer datasets.^19^ The analysis included a workflow of five steps:

1. Detection of the tissue area and removal of the background.
2. Splitting the WSI into smaller tiles (512 × 512 pixels) for subsequent analysis.
3. Nuclei segmentation to create binary masks.
4. Classification of nuclei as inflammatory or non-inflammatory.
5. Segmentation of inflammatory foci.

**Fig. 1:**
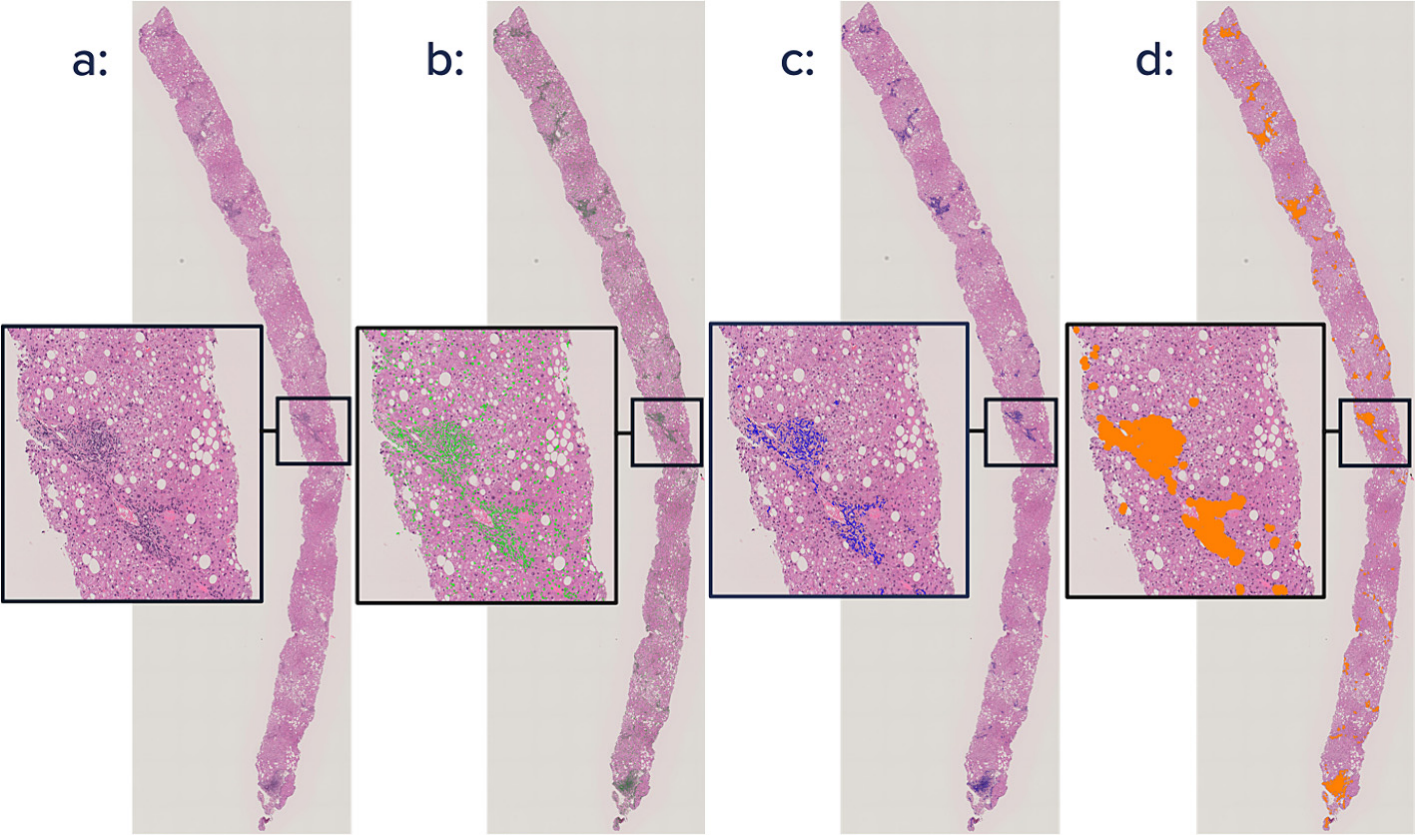
Digital image analysis workflow in a biopsy specimen with autoimmune hepatitis diagnosis (20× magnification). Mask detection was based on morphological analysis, size threshold, and color detection. Though the analysis was performed on the whole slice, a small region of interest has been highlighted to display details of the image processing algorithm (20× magnification). Once scanned and (a) the tissue area is detected and the background removed, (b) cell nuclei are then segmented (green). A binary mask is then applied to (c) classify nuclei as inflammatory or non-inflammatory (blue) after which (d) a mask is applied to the segmented inflammatory foci to identify regions with inflammation. This segmentation can then be used to assess inflammation density (ID: 7.4% H&E), and focal density (FD: 30.89 foci/mm^2^) for each case.

Steps 3–5 are presented in Fig. 1. Tissue area was detected by applying an edge detection filter to the grayscale version of the WSI. Areas with a high number of edges were separated using a threshold and marked as foreground (tissue) regions. Once tiled (into nonoverlapping 512 × 512 pixel segments), nuclei segmentation, using StarDist^23^ architecture, ensued and produced binary masks depicting the nuclei. Using pre-defined spatial proximity criteria, inflammatory nuclei were identified after which a disc shape kernel was applied to a binary mask of inflammatory nuclei to get a mask of regions with inflammation. The number of foci (morphologic clusters of inflammatory cells) was then counted using connected-component labeling, allowing for the quantification of the following scores:

(1) $$\text{Inflammation Density}\;\left(\mathrm{ID}\right)=\left(\text{Area of Inflammation}\right)/\left(\text{Area of Tissue}\right)$$

(2) $$\text{Focal Density}\left(\mathrm{FD}\right)=\text{(Number of Foci in total tissue)}/\text{(Total Area of Tissue)}$$

### Statistical analysis

Descriptive statistics were used to summarize cohort characteristics. Continuous variables were reported as mean and standard deviation (SD) with ranges reported where appropriate. Categorical variables were reported as frequency and percentage. Correlations between measurements were investigated using Spearman’s rank correlation coefficient (r_s_) with correlations greater than 0.60 considered strong.^17^

Patients were dichotomized into two clinically relevant groups according to histological inflammation grading. Those classified as having low-moderate disease had both portal and lobular inflammation grades ≤1, while those classed as having more advanced diseases (i.e., moderate-severe activity) had either portal or lobular inflammation grade ≥2. Comparisons between digital pathology measurements and disease categories were assessed using Wilcoxon rank sum tests. Moreover, comparisons between inflammation severity (low-moderate vs moderate/severe) were performed using independent samples *t*-test.

The ability of ID and FD to stratify cases between those with low-moderate and moderate-severe disease activity was estimated using the area under the receiver operating characteristic curve (AUC). For each metric (ID and FD), the optimal cut-off value was selected using Youden’s index.

Data were analyzed using the statistics package SciPy (version 1.10.0) in Python (version 3.8.6). Values of *p* < 0.05 were considered statistically significant. Case-wise deletion was performed where digital pathology images were missing.

## Results

### Patients and histological characteristics

Liver biopsies from 50 patients with biopsy confirmed AIH (55% female, with mean age 14 ± 3 years) undergoing routine liver biopsy as part of standard of care were included into this study. A summary of clinical and study participants demographic data alongside the traditional histopathology scores for portal and lobular inflammation is shown in Table 1. The mean biopsy length was 19.9 ± 9.4 mm (range: 8–42 mm) and included at least 10 portal tracts. Histological review showed that 54% had moderate-severe disease activity.

**Table 1:** Population demographics showing characteristics of the included autoimmune hepatitis population.

Characteristic	Overall cohort (*N* = 50)
Age	14 ± 3
Female sex (%)	55%
BMI (kg/m^2^)	20.8 ± 3.98
Biopsy length (mm)	19.9 ± 9.4
Blood serum tests	
ALT (IU/L)	136 ±287
AST (IU/L)	120 ± 279
Gamma globulins (%)	18.3 ± 5.4
IgG (g/L)	13.4 ± 4.7
GGT (IU/L)	59.6 ± 96.3
Albumin (g/L)	43.4 ± 4.3
Multiparametric MRI	
cT1 (ms)	834 ± 78
cT1 interquartile range (ms)	123 ± 35
Histology scores	
Portal inflammation	
Grade 0	7 (14%)
Grade 1	16 (32%)
Grade 2	17 (34%)
Grade 3	10 (20%)
Lobular inflammation	
Grade 0	20 (40%)
Grade 1	23 (46%)
Grade 2	4 (8%)
Grade 3	3 (6%)
Low-moderate disease activity	23 (46%)
Moderate-severe disease activity	27 (54%)

### Digital image analysis and inflammation

**Fig. 2:**
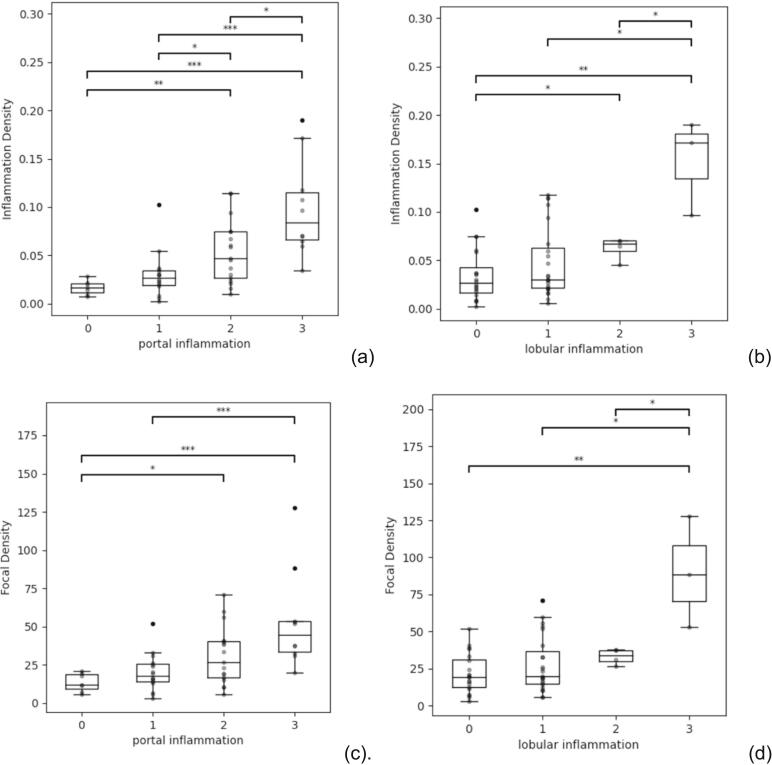
Distribution of digital pathology data across inflammatory activity histological scores. Relationship between inflammation density with (a) portal and (b) lobular inflammation, as well as focal density with (c) portal and (d) lobular inflammation are shown.

Distribution of ID and FD scores across histological grades is shown in Table 2. Both ID and FD increased significantly with inflammation grades (Table 2, Fig. 2). When comparing between disease severity, those with less-advanced disease (low-moderate inflammation) had significantly less ID (*p* < 0.001) compared to those with more advanced (moderate-severe) disease (2.6% vs 7.0%, respectively). Moreover, there were also significant differences (*p* < 0.001) in FD between disease severity groups (18.0 vs 39.1 foci/mm^2^) (Table 3).

**Table 2:** Summary of inflammation density (ID; % H&E), and focal density (FD; foci/mm^2^) results (±SD) for each grade of lobular and portal inflammation.

	Inflammation density	Focal density
*Lobular inflammation score*		
0	0.034 ± 0.027	21.633 ± 13.380
1	0.048 ± 0.037	27.698 ± 18.844
2	0.063 ± 0.012	32.995 ± 5.265
3	0.153 ± 0.049	89.621 ± 37.525

*Portal inflammation score*		
0	0.017 ± 0.007	13.526 ± 6.007
1	0.030 ± 0.023	19.957 ± 12.093
2	0.054 ± 0.033	30.841 ± 18.831
3	0.098 ± 0.050	53.226 ± 32.199

**Table 3:** Distribution of digital pathology data across disease categories of inflammation in autoimmune hepatitis (low-moderate and moderate-severe inflammation). Low-moderate: portal and lobular inflammation grades ≤1; moderate-severe: portal and lobular inflammation grades >2.

	Low-moderate	Moderate-severe	*p*-values
Inflammation density	0.026 ± 0.021	0.070 ± 0.045	<0.001
Focal density	18.000 ± 10.895	39.132 ± 26.428	<0.001

Portal and lobular inflammation correlated significantly with each other (R=0.58, *p* < 0.001). When assessing correlations between human read histological scores of portal and lobular inflammation, both scores correlated significantly with ALT (R=0.56, *p* < 0.001 and R=0.44, *p*=0.001, respectively) and AST (R=0.47, *p*=0.001 and R=0.40, *p*=0.004, respectively). Only portal inflammation correlated with IgG (R=0.34, *p*=0.003) and gamma globulins (R=0.41, *p*=0.004). Similarly, strong associations were observed between digital pathology and histology portal inflammation (0.58≤R≤0.7, *p* < 0.001) (Table 4). For histological lobular inflammation and digital pathology, moderate correlations were seen (R=0.4 for ID and R=0.36 for FD) with discrimination between grades 0 and 1 showing no differences (Table 4). In addition, when correlated with imaging markers, ID correlated significantly with cT1 (R=0.42, *p*=0.007), whereas FD correlated with both cT1 (R=0.40, *p*=0.01) and cT1 IQR (R=0.33, *p*=0.035).

**Table 4:** Correlations (R) between human read histological scores of portal and lobular inflammation as well as inflammation density (ID; % H&E), and focal density (FD; foci/mm^2^) with serum biochemical liver function test markers and imaging markers of liver health. All significant associations are highlighted in bold.

Biomarker	Lobular inflammation	Portal inflammation	Inflammation density	Focal density
*Histological scoring*				
Lobular inflammation	**–**	**0.58(<0.001)**	**0.40(0.004)**	**0.36(0.011)**
Portal inflammation	**0.58(<0.001)**	**-**	**0.69(<0.001)**	**0.59(<0.001)**
*Biochemical markers*				
ALT	**0.44(0.001)**	**0.56(<0.001)**	**0.55(<0.001)**	**0.55(<0.001)**
AST	**0.40(0.004)**	**0.47(0.001)**	**0.56(<0.001)**	**0.57(<0.001)**
GGT	0.16 (0.262)	**0.41(0.003)**	**0.46(0.001)**	**0.50(<0.001)**
IgG	0.26 (0.07)	**0.34(0.017)**	**0.43(0.002)**	**0.46(0.001)**
Gamma globulins	0.21 (0.146)	**0.41(0.004)**	**0.57(<0.001)**	**0.57(<0.001)**
*Imaging markers*				
cT1	**0.35 (0.008)**	**0.40 (0.007)**	**0.42 (0.007)**	**0.40 (0.010)**
cT1 IQR	−0.21 (0.17)	−0.15 (0.32)	0.31 (0.051)	**0.33 (0.035)**

**Fig. 3:**
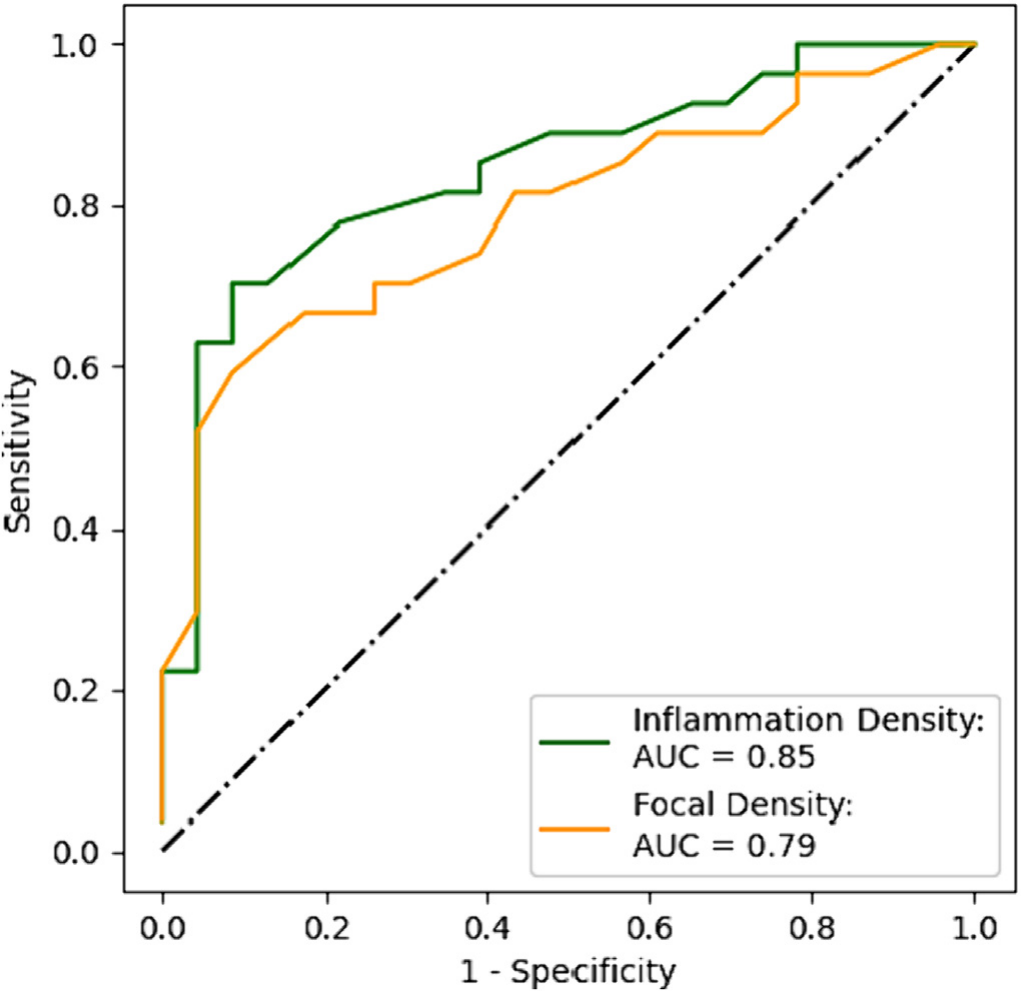
Diagnostic performance of digital pathology metrics for discriminating the presence of moderate/severe inflammation in autoimmune hepatitis cases. The green line corresponds to the inflammation density (ID) score (% H&E), and the orange line corresponds to the focal density (foci/mm^2^).

The AUC for the ID score for detecting moderate-severe inflammation (≥grade 2) was 0.85 (95% CI: 0.74–0.96) with a Youden’s index of 0.038, sensitivity of 0.70 and specificity of 0.91 (Fig. 3). Moreover, the associated NPV and PPV for this cut-off value were 0.72 and 0.90, respectively. When assessing the performance of the FD score, the AUC for detecting moderate-severe inflammation was 0.79 (95% CI: 0.67–0.92). The associated Youden’s index for FD was 30.97 with a sensitivity of 0.59, specificity of 0.91, NPV of 0.66 and PPV of 0.88 (Fig. 3).

### Digital pathology, biochemical markers, and imaging

When digital pathology scores (ID and FD) were correlated with imaging markers of disease activity (cT1 and cT1 IQR), significant relationships were seen between ID and cT1 as well as between FD and both cT1 and cT1 IQR (Table 4). Similarly, when compared to biochemical markers of liver function, both ID and FD correlated significantly with alanine transaminase (ALT), aspartate transaminase (AST), gamma-glutamyl transferase (GGT), Immunoglobulin G (IgG) and gamma globulins (Table 4). The strongest associations (R ≥ 0.55) between ID and FD were seen in ALT, AST, and gamma globulins.

## Discussion

Because of subjectivity, reproducibility and repeatability are two of the main issues plaguing classic histopathology today. Objective and repeatable quantification of liver histology can play a key role in overcoming/limiting these discrepancies. In this study, we investigated the use of digital pathology and image processing for the quantitative and morphometric assessment of inflammatory activity in AIH. Our findings showed that quantitative digital image analysis-derived parameters are ideally suited to differentiate between grades of inflammation, and therefore can support with stratification of disease severity.

Inflammation can vary substantially across liver tissue, ranging from lobular (used to assess the inflammatory foci in within the lobules), portal (used to assess the inflammation within the portal tracts), and periportal interface (used to assess the extension of inflammatory foci into the surrounding parenchyma).^10^ This variation may have an impact on the repeatability of grading between pathologists especially as there are a range of stains (e.g., H&E staining, Masson's trichrome staining, Periodic Acid Schiff staining, etc.) which can be used to assess inflammation. The high inter-observer variability between pathologists when assessing inflammation^7^ highlights the need for more objective methods to support standardisation of inflammation assessment. This is especially important as inflammation assessment forms a key part of chronic liver disease evaluation (at diagnosis, monitoring, and treatment response evaluation) and physician decision-making.

Quantitative digital pathology offers multiple opportunities to assess liver tissue regardless of the underlying cause of damage (including etiology, pattern of injury, and localization).^16^ Therefore, digital pathology lends itself as a useful technique to support pathologists in the assessment of tissue with variable regions of inflammation. Although color is considered one source of histopathological image variation when using H&E, similar to other digital pathology software’s using this staining,^9^^,^^26^ the algorithms used in this study were previously tested and validated using a wide range of stain qualities. When compared to other digital pathology software’s assessing inflammation,^15^ the current metrics performed similarly for the assessment of moderate-severe disease inflammatory activity,^15^ albeit with the advantage of using a simple and widely used staining method. By assessing tissue stained using commonly used stains in both clinical trial and clinical workflows,^5^^,^^22^ the software used in this study has far greater potential for clinical adoption than other computation inflammation measurement tools that require immunohistological staining such as CD45+^15^ or a rare type of microscopy like Second Harmonic Generation.^14^

Despite being an emerging tool, in the management of chronic liver disease such as hepatitis C and alcoholic liver disease, digital pathology imaging analyses are demonstrating increasing reliability as precise tools for quantitative histological assessment, continuous staging of hepatic fibrosis, and have shown prognostic utility in the prediction of adverse clinical outcomes.^11^^,^^25^ However, despite these advances, the quantification of inflammatory histologic activity is still lagging, and the understanding of the potential role of quantitative patterns of injury describing morphometric cell clustering is limited.^15^ The present study provides evidence that digital pathology-derived measurements of inflammation increase with both portal and lobular inflammation grades. More specifically, our findings show moderate correlation between both ID and FD and portal inflammation (0.59 ≤R≤ 0.69). AIH typically presents with inflammatory infiltrate located primarily in the portal tracts (portal inflammation), and thus, these findings reinforce the utility of simple computational algorithms based on imaging processing and expected morphological presentation. Furthermore, we observed correlation of lobular inflammation grades with these H&E staining metrics which are comparable to those reported in literature (0.36 ≤ R ≤ 0.40).^9^^,^^27^

Assessment of disease severity in AIH is typically described using the modified Hepatic Activity Index (mHAI).^10^ Additionally, definitions of full remission and subsequently decisions to withdraw treatment are also expressed using mHAI. Activity classification of inflammation severity (number of foci per × 10 field and portal inflammation) forms a key part of the mHAI score. Therefore, objective assessment of these key inflammation features, detected with quantitative digital image analysis metrics, can support whole tissue assessment and support more standardized definitions of disease severity. Our study showed, as expected, that when compared to their counterparts with low-moderate disease activity, those with moderate-severe disease had significantly higher focal density (foci per mm^2^) and inflammation density (inflammation coverage across the tissue). Moreover, when used to discriminate between low-moderate and moderate-severe disease activity, digital pathology metrics have good diagnostic performance (0.79 ≤AUC≤ 85).

This study had several strengths and some limitations. Firstly, we show the utility of digital pathology algorithms to support liver pathology assessment using simple widely used staining. Moreover, although our digital pathology metrics did not differentiate between lobular and portal inflammation, we investigated the association of each type of inflammation with the digital morphological assessment. This is important, as different diseases have varying inflammatory characteristics and thus assessment of both kinds of inflammation (portal and lobular) is vital if such techniques are to be clinically adopted. Although this is similar in nature to other analyses currently being used and investigated,^9^^,^^15^^,^^26^ it is possible that disease classification accuracy can be further enhanced by quantifying cellular infiltrates; future studies should explore this. A limitation to this study was that it was a cross-sectional, therefore, changes in the metrics over time during the disease course, or in response to treatment were not investigated. This evaluation will provide useful clinical information regarding the utility of these markers to support the evaluation of meaningful change, therefore, future studies (including clinical trials assessing pharmacotherapies) should investigate these changes. This is especially important as traditional grading systems may not be sufficient to detect small granular changes across the entire acquired liver tissue biopsy sample.^4^ Population bias could also be at play as only AIH patients were used in this study, therefore, future studies should investigate the utility of these quantitative markers in a wider range of inflammatory chronic liver diseases.

In summary, a major problem in histopathology is reproducibility due to inherent subjectivity of pathologist interpretation of the images. Quantitative digital pathology allows for repeatable, automated computational analysis of the morphological characteristics of hepatic inflammatory tissue. This assessment can provide quantitative metrics which can support pathologists with tissue assessment. Our findings showed that digital image analysis-derived parameters have good relationship with consensus scoring by expert pathologists. Accordingly, the development of inflammation and focal density, based on widely used staining methods, offers clear potential as a support tool to aid pathologists during the evaluation of chronic liver disease biopsies in clinical practice and clinical trials.

## Funding statement

This paper presents independent research funded by the Eureka Eurostars 2 Grant (E!10124)

## Ethics approval

This study received approval from the the Komisja Bioetyczna przy Instytucie “Pomnik-Centrum Zdrowia Dziecka” (11/KBE/2016).

## Data sharing

The data and analytic methods used in this study remain the property of the individual study sponsors. All deidentified participant data may be made available to other researchers upon request following permission, investigator support and following a signed data access agreement.

## Authors contributions

Study concept development: PS and KJ

Funding acquisition: PS, KJ, RB

Data collection: MP, WG, MW, PP, SC, EJ

Data analysis: AR, CL, KF, PA, ES

Manuscript drafting: ES, AR

Manuscript review and editing: KF, AR, ES, CL, KJ, PA, MW, SC, EJ, RB, PS, MP, WG

All authors contributed and approved the final manuscript.

## Declaration of competing interest

The members and employees of The Children's Memorial Health Institute declare no conflict of interest with this study. Perspectum Ltd is a privately funded commercial enterprise that develops medical devices to address unmet clinical needs, including LiverMultiScan. Perspectum is the sponsor of this study.

The authors declare the following financial interests/personal relationships which may be considered as potential competing interests:

Elizabeth Shumbayawonda reports financial support was provided by Perspectum Ltd. Abhishek Roy reports financial support was provided by Perspectum Ltd. Caitlin Langford reports financial support was provided by Perspectum Ltd. Paul Aljabar reports financial support was provided by Perspectum Ltd. Rajarshi Banerjee reports financial support was provided by Perspectum Ltd. Ken Fleming reports financial support was provided by Perspectum Ltd. Elizabeth Shumbayawonda reports a relationship with Perspectum Ltd. that includes: employment and equity or stocks. Abhishek Roy reports a relationship with Perspectum Ltd that includes: employment. Caitlin Langford reports a relationship with Perspectum Ltd that includes: employment. Paul Aljabar reports a relationship with Perspectum Ltd that includes: employment and equity or stocks. Rajarshi Banerjee reports a relationship with Perspectum Ltd that includes: employment and equity or stocks. Ken Fleming reports a relationship with Perspectum Ltd that includes: consulting or advisory.
